# Changes of C-reactive protein and Procalcitonin after four weeks of treatment in patients with pulmonary TB

**DOI:** 10.1016/j.jctube.2023.100348

**Published:** 2023-01-18

**Authors:** Marielle Leboueny, Anicet Christel Maloupazoa Siawaya, Loraine Daisy Josiane Bouanga, Ofilia Mvoundza Ndjindji, Amandine Mveang Nzoghe, Joel Fleury Djoba Siawaya

**Affiliations:** a Service Laboratoire, Centre Hospitalier Universitaire Mère-Enfant Fondation Jeanne EBORI BP. 212, Libreville, Gabon; b Unité de Recherches et de Diagnostics Spécialisés, Laboratoire National de Santé Publique, Avenue Felix Eboué, BP10 736 Libreville, Gabon; c Hôpital d'Instruction des Armées Omar Bongo Ondimba, Libreville, Gabon

**Keywords:** Tuberculosis, C-reactive protein (CRP), Procalcitonin (PCT), Biomarkers

## Abstract

**Objective:**

Tuberculosis (TB) remains a public health concern worldwide, affecting millions of people every year. Detailed characterization of disease pathophysiology is key to proper diagnosis, disease progression, or treatment follow-up and evaluation. The present study investigated C-reactive protein and Procalcitonin (PCT) as candidate markers of early treatment response and disease activity.

**Methods:**

**From** September to December 2019**,** 21 HIV-negative **consecutive** TB patients were recruited, within the setting of the Gabonese TB specialized hospital and the National Laboratory of Public Health, in a prospective study. CRP and PCT levels were measured by chemiluminescence at diagnosis and 4 weeks following the initiation of anti-TB treatment.

**Results:**

The mean concentration of CRP in TB patients was 114.7 mg/L (95 % CI: [83.8–145.6]) at diagnosis and 20.2 mg/L (95 % CI: [14.1–26.4]) 4 weeks following anti-TB treatment. The drop in CRP concentrations between diagnosis, and week 4 following anti-TB treatment showed was significant (p < 0.0001). The average concentration of PCT at the time of diagnosis was 0.3 ng/mL (95 % CI: [0.19–0.41]). PCT Concentration dropped below 0.05 ng/mL 4 weeks following the start of anti-TB treatment (p < 0.01).

**Conclusion:**

CRP and PCT are potential TB biomarkers, each, carrying important keys. If the drop in both proteins may indicate a significant reduction of the *Mtb* burden, the maintenance of CRP above the inflammation threshold could indicate the presence of residual bacilli. However, the clinical translation of the present finding will require more investigation.

## 1 Introduction

Tuberculosis (TB) continues to be a public health concern worldwide. An estimated 10 million people got active TB worldwide.[1] For 20 years the disease kills more than one (1) million people per year.[2] 1.5 million people died from TB in 2020.[1] Numerous studies have contributed to our current understanding of the complex biology of pulmonary tuberculosis and have subsequently provided evidence that immune or biochemical parameters involved in TB response could serve as diagnostic, disease activity, and progression biomarkers.[3], [4], [5], [6], [7] The search for TB biomarkers is ongoing.[8], [9] In the present study we investigated C-reactive protein (CRP) and Procalcitonin (PCT) as candidate markers for early TB disease activity.

C-reactive protein and Procalcitonin have been used as diagnostic markers of bacterial sepsis.[10], [11], [12] Although CRP has been previously investigated in TB, this is the first study looking at the potential value of PCT in tuberculosis disease.

## 2 Methods

We conducted a longitudinal prospective study, evaluating the change in CRP and PCT between diagnosis and the first month of the anti-TB standard treatment intensive phase. During this intensive phase all patients received isoniazid (INH), rifampin (RIF), pyrazinamide (PZA), and ethambutol (EMB) as recommended.[13].

Between September and December 2019, consecutive HIV-negative TB patients were recruited within the setting of the Gabonese TB specialized hospital and the National Laboratory of Public Health. TB disease was diagnosed by sputum smear and Xpert® MTB/RIF assay (GeneXpert, Cepheid – Europe).

For each patient, sputum smear tests were done and four (4) ml of blood was collected at diagnosis and one (1) month following the initiation of anti-TB therapy. Collected blood samples were transported for serum processing and analysis at the laboratory of the Libreville Mother and Child University Hospital. CRP levels were measured using the Cobas CRP test (on Cobas C111 analyzer, Roche - France) and PCT levels were measured using the VIDAS® B.R.A.H.M.S. PCT assay (Biomerieux - France). The data obtained were then analyzed using Graph Pad Prism Software.

### 2.1 Ethics

The research was done following Gabonese ethical guidelines and regulations, and approval was obtained from the Gabonese national ethics committee and registered under the number: PROT-N°0089/2019/PR/SG/CNER. Written informed consent, was obtained for all participants before they were enrolled in the study.

## 3 Results and discussion

A total of 21 HIV-negative TB patients were recruited (81 % men and 18 % women) aged between 28 and 57 years old (median age: 32 years). The Xpert® MTB/RIF assay showed that all patients had drug sensitive Mtb strains. 11 patients were lost during follow-up, and 10 were seen at month 1. Seven (7) patients were sputum negative after one (1) month of treatment, whereas, three (3) remained positive (Table 1).

**Table 1:** *Mycobacterium Tuberculosis* (Mtb) load, CRP, and PCT concentration at diagnosis and four (4) weeks after the initiation of anti-tuberculosis (anti-TB) standard treatment.

			**Diagnostic (pre-treatment)**			**After 4 Weeks of anti-TB treatment**		
**ID**	**Sex**	**Age**	**Bacilli load**	**CRP (mg/L)**	**PCT (ng/mL)**	**Bacilli load**	**CRP (mg/L)**	**PCT (ng/mL)**
P1	M	30	2+	115,42	0,17	0	7,22	<0,05
P2	M	34	3+	82,94	0,3	1+	19,1	<0,05
P3	M	30	2+	180,09	0,12	0	19,23	<0,05
P4	M	27	2+	98,34	0,32	0	17,4	<0,05
P5	M	48	1+	134	0,5	0	14,34	<0,05
P6	M	57	1+	176	0,27	0	34,18	<0,05
P7	F	41	3+	43,78	0,27	1+	17,75	<0,05
P8	M	28	2+	90,14	0,5	0	14,89	<0,05
P9	M	28	2+	85,83	0,5	1+	34,45	<0,05
P10	M	51	2+	140,57	0,07	0	23,96	<0,05
P11	M	41	1+	142,5	1,15	**No follow-up**		
P12	F	35	1+	99,92	0,05			
P13	F	28	2+	70,08	0,05			
P14	M	32	3+	107,7	0,07			
P15	M	27	2+	132,73	0,05			
P16	F	28	3+	184	0,17			
P17	M	34	2+	57,23	0,05			
P18	M	41	3+	189,94	0,07			
P19	M	38	2+	85,31	0,12			
P20	M	28	3+	14,34	0,06			
*P*21	M	28	3+	44,08	0,06			

**Fig. 1:**
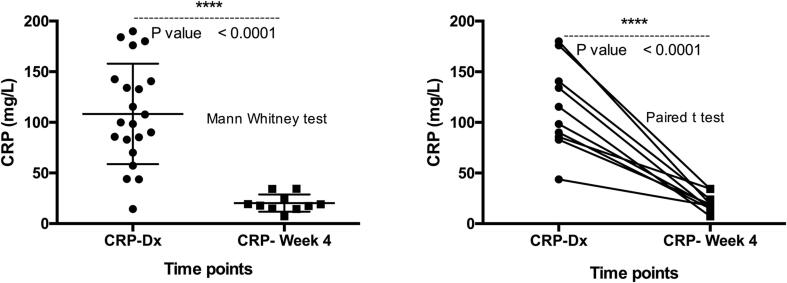
The concentration of CRP at diagnosis and 4 weeks after the initiation of treatment. (a) Unpaired (Mann Whitney) *t*-test; (b) illustration of the paired *t*-test. The statistical significance was defined as p < 0.05.

**Fig. 2:**
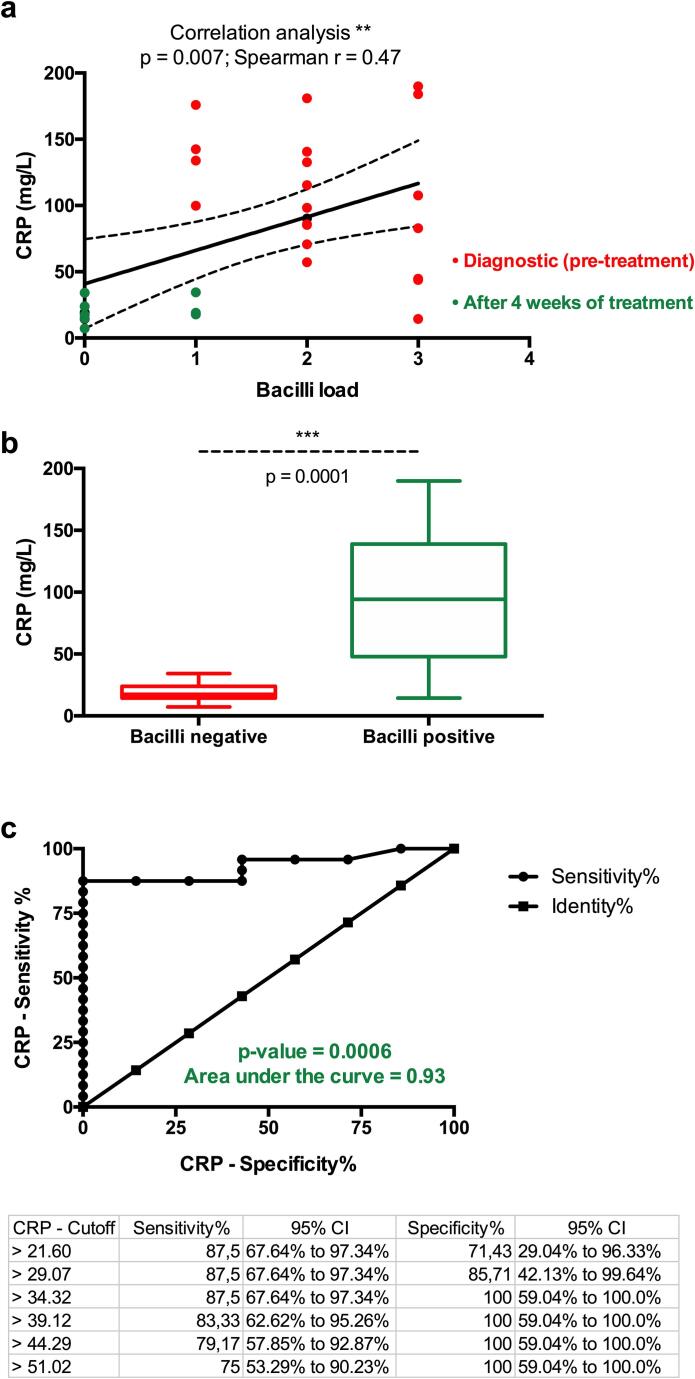
(a) Correlation between bacilli count and the circulating concentration of CRP in patients (r = 0.47; p = 0.007). (b) Bacilli-positive patients had a significantly higher level of CRP (p < 0.0005). (c) The receiving operator curve (ROC) analysis of CRP as indicator of bacilli-positivity (AUC: 0.93; p = 0.0006).

The median and the mean levels of CRP in TB patients at diagnosis were respectively 99.92 mg/L (IQR: 71) and 108.3 mg/L (95 % CI: [83.8–130.9]). This level corresponded to the levels observed in bacterial infection.[14], [15] 4 weeks after the initiation of treatment, CRP median and mean concentration dropped respectively to 18.43 mg/L (IQR: 11.77) and 20.25 mg/L ((95 % CI: [14.1–26.4]). Both non-parametric and parametric comparisons of the circulating CRP concentrations between diagnosis, and 4 weeks following anti-TB treatment showed that the drop in CRP was significant (p < 0.0001) (Fig. 1). Further analysis showed a significant correlation between bacilli count or presence and the circulating concentration of CRP in patients (p = 0.007) (Fig. 2a). Bacilli-positive patients had a significantly higher level of CRP (p < 0.0001) (Fig. 2b). The receiving operator curve (ROC) analysis confirmed that CRP might be useful in discriminating bacilli-positive patients (AUC: 0.93; p = 0.0006) (Fig. 2c). Although the levels of CRP decreased drastically after one (1) month of treatment, the levels remained above the inflammation threshold, [16], [17] even in patients with negative sputum smears. This may be the reflection of the underlining activity of non-sterilized bacilli, as an optimal *Mycobacterium Tuberculosis* (*Mtb*) sterilization requires a 6 months regimen.

Also, CRP being an opsonizing factor, its production should reflect bacilli presence. It has been reported that a high level of CRP after anti-TB drug therapy was associated with a delay in bacilli negativation.[18] Although not investigated here, others have shown the return of CRP to its normal levels after 6 months. [19] Moreover, studies have shown CRP to have a good screening performance for active pulmonary tuberculosis in both HIV-positive and negative patients. [4], [20], [21], [6] This comfort CRP as an indicator of disease activity.

**Fig. 3:**
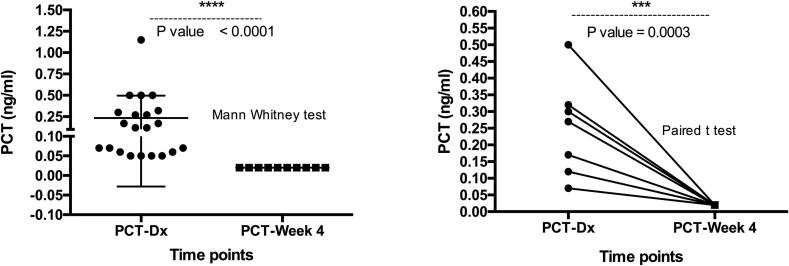
The concentration of Procalcitonin at diagnosis and 4 weeks after the initiation of treatment. (a) Unpaired (Mann Whitney) t-test; (b) illustration of the paired t-test. The statistical significance was defined as p < 0.05.

**Fig. 4:**
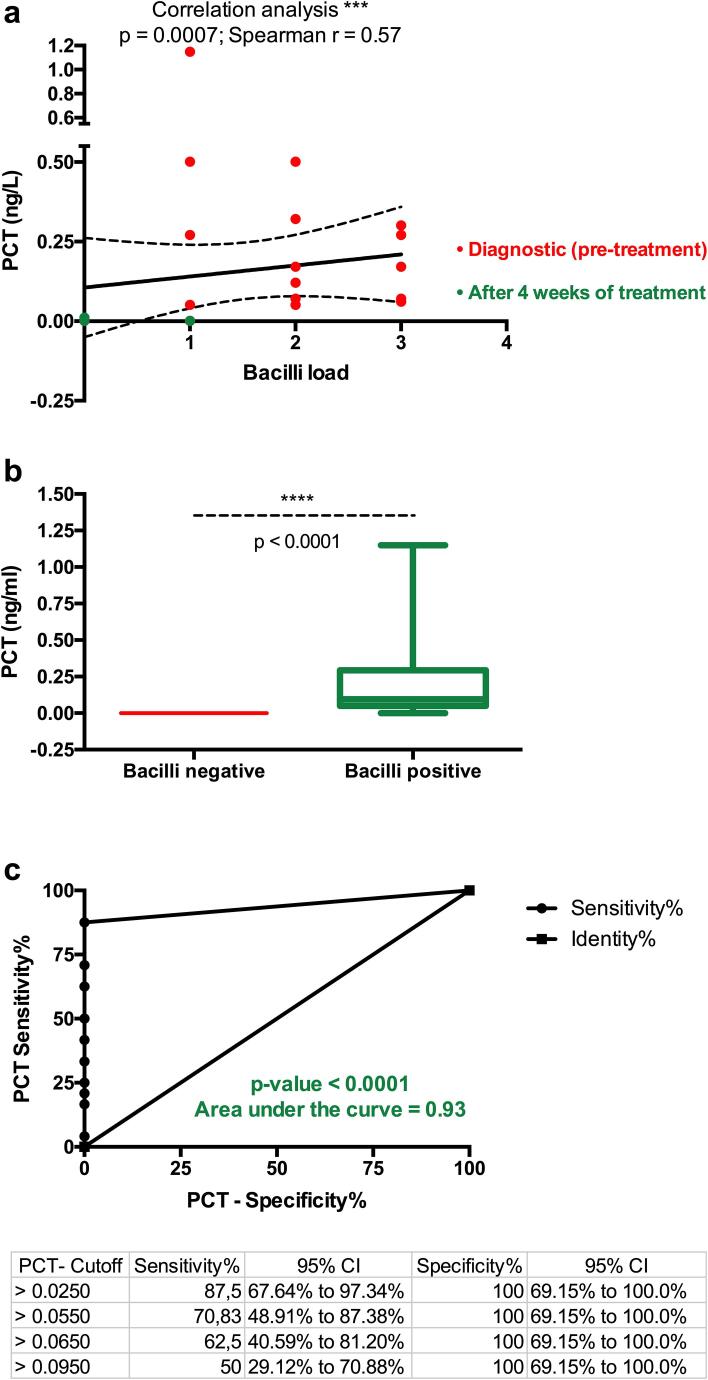
(a) Correlation between bacilli count and the circulating concentration of PCT in patients (r = 0.57; p = 0.0007). (b) Bacilli-positive patients had a significantly higher level of PCT (p < 0.0001). (c) The receiving operator curve (ROC) analysis of PCT as indicator of bacilli-positivity (AUC: 0.93; p < 0.0001).

PCT has been investigated for different conditions and diseases. [10], [11], [22], [23], [24], [25], [26], [27], [28], [29], [30], [31], [32] However, this is one of the two studies investigating the level of PCT in tuberculosis. At diagnosis, all TB patients had their procalcitonin (PCT) concentrations between 0.05 and 1.15 ng/ml (median level: 0.12 ng/ml (IQR: 0.25)), which is above the normal range. 95 % (20 out of 21) of patients had a level of PCT corresponding to localized infection (0.05–0.5 ng/ml). Only one patient had a level of PCT in the sepsis range (1.15 ng/ml). The average concentration of PCT at the time of diagnosis was 0.3 ng/mL (95 % CI: [0.19–0.41]). In all patients seen after 4 weeks of anti-TB treatment, PCT concentration dropped below 0.05 ng/mL (which is the normal range). Both non-parametric and parametric analysis showed that the level of PCT at diagnosis is significantly higher than its level 4 weeks following anti-TB treatment (p < 0.0001 and p = 0.003 respectively) (Fig. 3). Correlation analysis showed that the circulating concentration of PCT correlates significantly with bacilli count (p = 0.0007) (Fig. 4a). Bacilli-positive patients had a significantly higher level of PCT (p < 0.0001) (Fig. 4b). The ROC analysis also showed that PCT might be useful in discriminating bacilli-positive patients (AUC: 0.93; p < 0.0001) (Fig. 4c).

It has been suggested that the concept of predictive models using combinations of non-specific host markers may be valid. [4], [9], [33] The challenge is to identify the strongest markers that will contribute to such models. [8], [34] Reports showed that PCT levels correlate with pneumonia severity and prognosis, improving the prognostic power of severity scoring systems. [35], [36], [37] Also, PCT is being used not only as a marker of bacterial sepsis but also as an indicator to guide the decision to initiate and stop antibiotic treatment. [38], [39] Furthermore, here we showed that PCT and CRP dropped with *Mycobacterium Tuberculosis* (*Mtb*) load (Table 1) and represent candidate markers for active TB disease and treatment response.

The low number of patients and the high rate of patients lost to follow-up characterized the present study. Therefore, despite the observed statistically, significant correlations between the selected markers (PCT and CRP) and bacilli load, the translation of these results into clinical use will require more investigations.

**In conclusion,** CRP and PCT are potential active TB biomarkers, that may carry important information on inflammation and bacilli load. However, further investigation is needed to establish PCT as a marker of TB treatment response and disease prognosis. Also, the value of associating PCT and CRP for the diagnosis of TB severity and prognosis needs to be investigated.

## Declaration of Competing Interest

The authors declare that they have no known competing financial interests or personal relationships that could have appeared to influence the work reported in this paper.

## References

[1] Global tuberculosis report 202Geneva: World Health Organization; 2021.

[2] Global Tuberculosis Report 2020. Geneva: World Health Organization; 2020.

[3] Djoba Siawaya J.F., Bapela N.B., Ronacher K., Beyers N., van Helden P., Walzl G. (2008). Differential expression of interleukin-4 (IL-4) and IL-4 delta 2 mRNA, but not transforming growth factor beta (TGF-beta), TGF-beta RII, Foxp3, gamma interferon, T-bet, or GATA-3 mRNA, in patients with fast and slow responses to antituberculosis treatment. Clin Vaccine Immunol.

[4] Djoba Siawaya J.F., Bapela N.B., Ronacher K., Veenstra H., Kidd M., Gie R. (2008). Immune parameters as markers of tuberculosis extent of disease and early prediction of anti-tuberculosis chemotherapy response. J Infect.

[5] Djoba Siawaya J.F., Ruhwald M., Eugen-Olsen J., Walzl G. (2007). Correlates for disease progression and prognosis during concurrent HIV/TB infection. Int J Infect Dis.

[6] Chegou N.N., Sutherland J.S., Malherbe S., Crampin A.C., Corstjens P.L., Geluk A. (2016). Diagnostic performance of a seven-marker serum protein biosignature for the diagnosis of active TB disease in African primary healthcare clinic attendees with signs and symptoms suggestive of TB. Thorax.

[7] Chegou N.N., Sutherland J.S., Namuganga A.-R., Corstjens P.L., Geluk A., Gebremichael G. (2018). Africa-wide evaluation of host biomarkers in QuantiFERON supernatants for the diagnosis of pulmonary tuberculosis. Sci Rep.

[8] Walzl G., Ronacher K., Hanekom W., Scriba T.J., Zumla A. (2011). Immunological biomarkers of tuberculosis. Nat Rev Immunol.

[9] Ronacher K., Chegou N.N., Kleynhans L., Djoba Siawaya J.F., du Plessis N., Loxton A.G. (2019). Distinct serum biosignatures are associated with different tuberculosis treatment outcomes. Tuberculosis (Edinb).

[10] Alejandre C., Guitart C., Balaguer M., Torrús I., Bobillo-Perez S., Cambra F.J. (2021). Use of procalcitonin and C-reactive protein in the diagnosis of bacterial infection in infants with severe bronchiolitis. Eur J Pediatr.

[11] Blouin A.G., Hsu M., Fleisher M., Ramanathan L.V., Pastores S.M. (2020). Utility of procalcitonin as a predictor of bloodstream infections and supportive modality requirements in critically ill cancer patients. Clin Chim Acta.

[12] Anush M.M., Ashok V.K., Sarma R.I., Pillai S.K. (2019). Role of C-reactive Protein as an Indicator for Determining the Outcome of Sepsis. Indian J Crit Care Med.

[13] Nahid P, Dorman SE, Alipanah N, Barry PM, Brozek JL, Cattamanchi A, Chaisson LH, Chaisson RE, Daley CL, Grzemska M, Higashi JM, Ho CS, Hopewell PC, Keshavjee SA, Lienhardt C, Menzies R, Merrifield C, Narita M, O'Brien R, Peloquin CA, Raftery A, Saukkonen J, Schaaf HS, Sotgiu G, Starke JR, Migliori GB, Vernon A. Official American Thoracic Society/Centers for Disease Control and Prevention/Infectious Diseases Society of America Clinical Practice Guidelines: Treatment of Drug-Susceptible Tuberculosis. Clin Infect Diseas 2016;**63**:e147-e195. doi: 10.1093/cid/ciw376. 10.1093/cid/ciw376 PMC6590850 27516382

[14] Haran J.P., Beaudoin F.L., Suner S., Lu S. (2013). C-reactive protein as predictor of bacterial infection among patients with an influenza-like illness. Am J Emerg Med.

[15] Escadafal C, Incardona S, Fernandez-Carballo BL, Dittrich S. The good and the bad: using C reactive protein to distinguish bacterial from non-bacterial infection among febrile patients in low-resource settings. *BMJ Glob Health* 2020;**5** doi: 10.1136/bmjgh-2020-002396. 10.1136/bmjgh-2020-002396 PMC7259834 32467355

[16] WHO/NMH/NHD. C-reactive protein concentrations as a marker of inflammation or infection for interpreting biomarkers of micronutrient status. *VMNIS;* 2014.

[17] Escadafal C, Incardona S, Fernandez-Carballo BL, Dittrich S. The good and the bad: using C reactive protein to distinguish bacterial from non-bacterial infection among febrile patients in low-resource settings. *BMJ Glob Health* 2020;**5**:e002396. doi: 10.1136/bmjgh-2020-002396. 10.1136/bmjgh-2020-002396 PMC7259834 32467355

[18] Kwas H., Guermazi E., Zendah I., Ben Jemia E., Khattab A., Khouaja I. (2015). C-reactive protein and pulmonary tuberculosis: What correlation with disease severity. Eur Respir J.

[19] Bajaj G., Rattan A., Ahmad P. (1989). Prognostic value of 'C' reactive protein in tuberculosis. Indian Pediatr.

[20] Ciccacci F., Welu B., Ndoi H., Karea I., Orlando S., Brambilla D. (2021). High-sensitivity C-reactive protein in HIV care: Tuberculosis diagnosis and short-term mortality in a cohort of Kenyan HIV patients in the DREAM programme. Int J Infect Dis.

[21] Meca A.D., Turcu-Stiolica A., Bogdan M., Subtirelu M.S., Cocoș R., Ungureanu B.S. (2022). Screening performance of C-reactive protein for active pulmonary tuberculosis in HIV-positive patients: A systematic review with a meta-analysis. Front Immunol.

[22] Abu Rahma M.Z., Mahran Z.G., Shafik E.A., Mohareb D.A., Abd El-Rady N.M., Mustafa M.A. (2021). The Role of Serum Procalcitonin Level as an Early Marker of Ascitic Fluid Infection in Post Hepatitic Cirrhotic Patients. Antiinflamm Antiallergy Agents Med Chem.

[23] Ahn J.H., Cho Y.S., Cho G.C. (2020). Elevated procalcitonin levels in patients with acetaminophen intoxication: two case reports: A CARE-compliant article. Medicine (Baltimore).

[24] Alessandri F., Pugliese F., Angeletti S., Ciccozzi M., Russo A., Mastroianni C.M. (2020). Procalcitonin in the Assessment of Ventilator Associated Pneumonia: A Systematic Review. Adv Exp Med Biol.

[25] Azzini AM, Dorizzi RM, Sette P, Vecchi M, Coledan I, Righi E, Tacconelli E. A 2020 review on the role of procalcitonin in different clinical settings: an update conducted with the tools of the Evidence Based Laboratory Medicine. *Ann Transl Med* 2020;**8**:610. doi: 10.21037/atm-20-1855. 10.21037/atm-20-1855 PMC7290560 32566636

[26] Bailey K.L., Murphy P.J., Lineberry O.K., Haack M.R., Dickinson J.D., Kalil A.C. (2020). Procalcitonin predicts the severity of cystic fibrosis pulmonary exacerbations and readmissions in adult patients: a prospective cohort study. J Investig Med.

[27] Bassetti M., Russo A., Righi E., Dolso E., Merelli M., D'Aurizio F. (2020). Role of procalcitonin in predicting etiology in bacteremic patients: Report from a large single-center experience. J Infect Public Health.

[28] Bishnoi N.K., Singh N., Sharma R. (2020). A prospective observational study to evaluate serum Procalcitonin as a bio marker of sepsis in critically ill patients and it's correlation with their clinicoetiological profi le. J Assoc Physicians India.

[29] Blot M., Chavanet P., Piroth L. (2020). Procalcitonin to Distinguish Viral From Bacterial Origin of Pneumonia: No Premature Conclusion!. Clin Infect Dis.

[30] Bontekoe J, Bansal V, Lee J, Syed M, Hoppensteadt D, Maia P, Walborn A, Liles J, Vasaiwala S, Fareed J. Procalcitonin as a Marker of Comorbid Atrial Fibrillation in Chronic Kidney Disease and History of Sepsis. *Clin Appl Thromb Hemost* 2020;**26**:1076029620932228. doi: 10.1177/1076029620932228. 10.1177/1076029620932228 PMC7427007 32539447

[31] Busch A., Jager M., Engler H., Haversath M., Bielefeld C., Landgraeber S. (2020). Is Procalcitonin (PCT) a reliable biomarker for preoperative diagnosing of low grade periprosthetic joint infection? A prospective study. BMC Musculoskelet Disord.

[32] Cancela-Nieto M.G., Sanchez-Sobrino P., Velogarcia A. (2020). Procalcitonin as a marker of medullary thyroid carcinoma. Minerva Endocrinol.

[33] Djoba Siawaya J.F., Beyers N., van Helden P., Walzl G. (2009). Differential cytokine secretion and early treatment response in patients with pulmonary tuberculosis. Clin Exp Immunol.

[34] Walzl G., Ronacher K., Djoba Siawaya J.F., Dockrell H.M. (2008). Biomarkers for TB treatment response: challenges and future strategies. J Infect.

[35] Muller F., Christ-Crain M., Bregenzer T., Krause M., Zimmerli W., Mueller B. (2010). Procalcitonin levels predict bacteremia in patients with community-acquired pneumonia: a prospective cohort trial. Chest.

[36] Naderi H.R., Sheybani F., Sarvghad M., Nooghabi M.J. (2015). Can Procalcitonin Add to the Prognostic Power of the Severity Scoring System in Adults with Pneumonia?. Tanaffos.

[37] Nobre V., Borges I. (2016). Nucleo Interdisciplinar de Investigacao em Medicina I. Prognostic value of procalcitonin in hospitalized patients with lower respiratory tract infections. Rev Bras Ter Intensiva.

[38] Daubin C., Valette X., Thiollière F., Mira J.-P., Hazera P., Annane D. (2018). Procalcitonin algorithm to guide initial antibiotic therapy in acute exacerbations of COPD admitted to the ICU: a randomized multicenter study. Intensive Care Med.

[39] Brechot N., Hekimian G., Chastre J., Luyt C.E. (2015). Procalcitonin to guide antibiotic therapy in the ICU. Int J Antimicrob Agents.

